# Experimental Researches on Cicatrization in Blood-vessels after Ligature

**Published:** 1885-07

**Authors:** 


Experimental Researches on Cicatrization in Blood-vessels after Ligature. *By* Professor N. Senn, m. d., *of Milwaukee, Wisconsin. Extracted from the Transactions of the American Surgical Association, vol. I. 1884.*

Professor Senn's recent valuable contribution to our knowledge of "Cicatrization in Blood-vessels After Ligature" has attracted so much attention at home and abroad that we append, at this late day, his conclusions:

A careful examination of the literature on the subject of this paper, as well as the results of my own investigations, warrant me in submitting for your further consideration and discussion the following conclusions:—

I. All operations on blood-vessls should be done under antiseptic precautions.

II. The aseptic catgut ligature is the safest and most reliable agent in securing provisional and definitive closure of blood-vessels.

III. A thrombus after ligature is an accidental formation which never undergoes organization and takes no active part in the obliteration of a vessel.

IV. The intra-vascular or definitive cicatrix is the exclusive product of connective tissue and endothelial proliferation.

V. Permanent obliteration in arteries takes place in from four to seven days, in veins in from three to four days.

VI. In ligating vessels in aseptic wounds the vessel sheath can be opened freely without compromising the integrity of the vessel tunics, and such procedure renders the operation safer and easier of execution.

VII. The double aseptic catgut ligature should be preferred to the single ligature in ligating large arteries in their continuity near a collateral branch, and should always be employed in all operations of tying varicose veins in their continuity as the safest and most effective measure in producing definite obliteration.

We are not able to fully agree with Professor Senn in *all* his conclusions, nor do we deem them entirely justified either by "a careful examination of the literature on the subject" or the results of his own investigations. During the discussion of his paper, probable sources of error in the experiments and fallacies in argument were pointed out by Dr. Donald Maclean, of Detroit, Dr. Henry F. Campbell, of Augusta, Dr.

E. H. Gregory, of St. Louis, Dr. J. S. Billings, United States Army, Dr. B. A. Watson, of Jersey City, and Dr. C. B. Nancrede, of Philadelphia.

The bibliography is not complete. The researches of French surgeons have received less attention than they merit, while the important investigations of Dr. O. F. Shakspeare, and B. Alexander Randall, of Philadelphia, are scarcely more than mentioned.

We are of the opinion that a large part of the value of the paper lies in the stimulation imparted to original research,—a field, with whose topography American surgeons are too little acquainted.

Professor Senn is entitled to the gratitude of the profession for this, as for numerous other meritorious contributions to surgical literature. W. W. J.

